# Kun-Dan Decoction Ameliorates Insulin Resistance by Activating AMPK/mTOR-Mediated Autophagy in High-Fat Diet-Fed Rats

**DOI:** 10.3389/fphar.2021.670151

**Published:** 2021-05-28

**Authors:** Zuqing Su, Kexue Zeng, Bing Feng, Lipeng Tang, Chaoyue Sun, Xieqi Wang, Caiyun Li, Guangjuan Zheng, Ying Zhu

**Affiliations:** Guangdong Provincial Hospital of Chinese Medicine, The Second Clinical College of Guangzhou University of Chinese Medicine, Guangzhou, China

**Keywords:** metabolic syndrome, insulin resistance, Kun-Dan decoction, AMPK/mTOR-mediated autophagy, network pharmacology

## Abstract

**Background:** Metabolic syndrome is characterized by central obesity, hyperglycemia and hyperlipidemia. Insulin resistance is the leading risk factor for metabolic syndrome. Kun-Dan decoction (KD), a traditional Chinese medicine, has been applied to treat patients with metabolic syndrome for over ten years. It is increasingly recognized that autophagy deficiency is the key cause of metabolic syndrome. Therefore, we aimed to explore whether KD can activate autophagy to improve metabolic syndrome.

**Methods:** Network pharmacology was used to explore the underlying mechanism of KD in the treatment of metabolic syndrome. The high-fat diet-fed rats and oleic acid-induced LO2 cells were employed in our study. Oral glucose tolerance test and insulin tolerance test, obesity and histological examination, serum cholesterol, triglyceride, low-density lipoprotein cholesterol (LDL-C), high-density lipoprotein cholesterol (HDL-C), homeostasis model assessment of insulin resistance (HOMA-IR) and insulin sensitivity in high-fat diet-fed rats were analyzed. Furthermore, the protein expressions of adenosine 5'-monophosphate (AMP)-activated protein kinase (AMPK), phospho-AMPK, mammalian target of rapamycin (mTOR), phospho-mTOR, p62, autophagy related protein (Atg) 5, Atg7, Atg12, Atg13, Atg16L1 and microtubule-associated protein 1A/1B-light chain 3 (LC3)-Ⅱ/Ⅰ were examined in rats and LO2 cells. Moreover, autophagy activator rapamycin and inhibitor 3-methyladenine, and small interfering RNA against Atg7 were utilized to verify the role of autophagy in the treatment of metabolic syndrome by KD in oleic acid-induced LO2 cells.

**Results:** Results from network pharmacology indicated that targeted insulin resistance might be the critical mechanism of KD in the treatment of metabolic syndrome. We found that KD significantly suppressed obesity, serum cholesterol, triglyceride and LDL-C levels and increased serum HDL-C level in high-fat diet-fed rats. Furthermore, KD enhanced insulin sensitivity and attenuated HOMA-IR in high-fat diet-fed rats. Western blot showed that KD could enhance autophagy to increase the insulin sensitivity of high-fat diet-fed rats and oleic acid-induced LO2 cells. Furthermore, 3-methyladenine and small interfering RNA against Atg7 could reverse the protective effect of KD on LO2 cells. However, rapamycin could cooperate with KD to enhance autophagic activation to increase insulin sensitivity in LO2 cells.

**Conclusion:** The induction of autophagy may be the major mechanism for KD to improve insulin resistance and metabolic syndrome.

## Introduction

Metabolic syndrome is characterized by hyperglycemia, hyperlipidemia and central obesity, which increases the risk of various diseases including cardiovascular disease, diabetes, non-alcoholic fatty liver and cancer (Lent-Schochet et al., 2019). Latest epidemiological data show that the global prevalence of metabolic syndrome exceeds 20% (Tahereh et al., 2020). Due to the lack of potent pharmacotherapy, the rising prevalence of metabolic syndrome poses a huge threat to human health worldwide. Nowadays, it is commonly accepted that unhealthy eating habits, physical inactivity and sedentariness play pivotal roles in the pathogenesis of metabolic syndrome (Liu et al., 2015). Of note, there is a consensus that insulin resistance is the leading cause of metabolic syndrome. Unhealthy eating habits, especially high-fat diets, initially induce hepatic insulin resistance, followed by adipose and muscle insulin resistance (Gao et al., 2010). Moreover, clinically, insulin resistance has occurred many years before the diagnosis of metabolic syndrome. Therefore, targeting insulin resistance is conducive to the prevention and treatment of metabolic syndrome (Shaodong, 2013).

Autophagy, a conservative catabolic process, can degrade excessive fatty acids, damaged cell structures and organelles in the lysosome to maintain cellular energy homeostasis (Vargas et al., 2017; Su et al., 2019). Amino acids and other small molecules produced by autophagic degradation are sent back to the cytoplasm for energy production (Noboru et al., 2010; Cerri and Blandini, 2018; Su et al., 2019). Accumulating evidence has shown that autophagy is involved in many physiological and pathological processes including metabolic syndrome, cardiovascular, respiratory, neurodegenerative and metabolic diseases (Hyejin et al., 2018; Pierzynowska et al., 2018; Racanelli et al., 2018; Ren and Zhang, 2018). Accordingly, these evidences suggest that the activation of autophagy may be beneficial for the prevention and treatment of metabolic syndrome.

Recently, given the relative safety and multiple beneficial effects, more and more researchers are searching for medicinal and edible herbs as complementary and alternative medicines. More and more medicinal and edible herbs have shown benefits to insulin resistance and metabolic syndrome. Kun-Dan (KD) consists of *Atractylodes macrocephala* Koidz., *Crataegus pinnatifida* Ege., *Citrus medica* L. var. *Sarcodactylis* Swingle, *Cassia obtusifolia* L. and *Ecklonia kurome* Okam. (Table 1) and has been used to treat patients with metabolic syndrome for over ten years. Our previous studies have shown that KD can significantly inhibit the levels of serum leptin, free fatty acids, tumor necrosis factor (TNF)-α and plasminogen activator inhibitor-1, and increase the expression of serum adiponectin in rats with metabolic syndrome (Guangjuan et al., 2014). Reports have demonstrated that TNF-α can inhibit the activation of phosphoinositide 3-kinases (PI3K)-AKT signaling pathway to induce insulin resistance (Khodabandehloo et al., 2015). Whereas adiponectin can enhance the activation of PI3K-AKT signaling pathway to increase insulin sensitivity (Kobashi et al., 2005). However, the molecular mechanism of KD against metabolic syndrome is not clearly elucidated. Latest evidence indicates that targeting autophagy is a promising treatment for metabolic syndrome (Hyejin et al., 2018). Accordingly, we seek to explore the role of autophagy in the treatment of metabolic syndrome by KD.

**TABLE 1:** Components of Kun-Dan.

Species name	Medicinal part	Weight (g)	Voucher number
Asteraceae; *Atractylodes macrocephala Koidz*.	Rhizome	12	KD01AMK
Rosaceae; *Crataegus pinnatifida* Ege.	Fruit	12	KD02CPE
Rutaceae; *Citrus medica* L. var. *Sarcodactylis* Swingle	Fruit	9	KD03CSS
Leguminosae; *Cassia obtusifolia* L.	Seed	15	KD04COL
Laminariaceae; *Ecklonia kurome* Okam.	Thallus	12	KD05EKO

In this study, we have found that KD can inhibit insulin resistance, obesity, hyperglycemia and hyperlipidemia of high-fat diet-fed rats. KD is also available to enhance insulin sensitivity of insulin-resistant LO2 cells. Mechanistically, the induction of autophagy is associated with the treatment of insulin resistance and metabolic syndrome by KD. This research allows us to better understand the role of autophagy in the treatment of metabolic syndrome by herbal medicine, and also provides theoretical support for dietary therapy.

## Materials and Methods

### KD Chemical Compounds and Metabolic Syndrome-Related Target Screening

The Traditional Chinese Medicine Systems Pharmacology (TCMSP) database and Traditional Chinese Medicine Integrative database (TCMID) were applied to collect the chemical ingredients of KD (Wei et al., 2019). Finally, 65 chemical ingredients were obtained, and ingredient-related targets were predicted using Drugbank and SwissTargetprediction databases (Wei et al., 2019; Liu et al., 2020).

The metabolic syndrome-related targets were screened by Therapeutic Target Database (TTD), Online Mendelian Inheritance in Man (OMIM) and DisGeNET databases (Liu et al., 2020). Eventually, coexistent targets between chemical ingredients and disease were screened as KD-related targets for metabolic syndrome.

### Network Construction

Based on the identified ingredients and targets of KD, the interaction network between compounds and targets was established by Cytoscape 3.7.2 software. The protein-protein interactions (PPI) were analyzed by String (https://string-db.org/, version 11.0). The protein’s topology analysis was executed by Cytoscape 3.7.2 with the plugin tool “CytoNCA” (Li et al., 2021).

### Gene Ontology and KEGG Enrichment Analysis

For the better understanding of underlying biological process, the Gene Ontology (GO) and Kyoto Encyclopedia of Genes and Genomes (KEGG) enrichment analyses were carried out with R/Bioconductor statistical analysis language and software (Haberman et al., 2013).

### Preparation of KD

The medicine herbals in KD were purchased from Guangdong Provincial Hospital of Chinese Medicine (Guangdong, China), and authenticated by Prof. Guangjuan Zheng. Voucher specimens were deposited in the research laboratory of herbal pharmacology of Guangdong Provincial Hospital of Chinese Medicine (Table 1). Briefly, *Atractylodes macrocephala* Koidz., *Crataegus pinnatifida* Ege., *Citrus medica* L. var. *Sarcodactylis* Swingle, *Cassia obtusifolia* L. and *Ecklonia kurome* Okam. were extracted twice in boiling water for 30 min each time. The extraction was filtered and concentrated in a rotary evaporator under reduced pressure. Ultimately, dry powder was manufactured by a freeze dryer at a relatively low temperature condition (−80°C).

### High Performance Liquid Chromatography Analysis

Qualitative analysis of KD was carried out by an Agilent 1260 high performance liquid chromatography with a G1315C DAD detector, a G1311B pump, a G1313A automatic sampler and a G1316A thermostatic column compartment (Agilent, California, United States). The working conditions were optimized and established as follows: column: ZORBAX SB-C18 (4.6 × 250 mm, 5 μm); mobile phase: water (A) and acetonitrile (B), a gradient mode (0–8 min, 95% A; 8–19 min, 95% A → 60% A; 19–25 min, 60% A → 24% A; 25–36 min, 24% A → 21% A); detection: UV, 220 nm; flow-rate: 0.8 ml/min. 0.3 g KD powder was dissolved in 25 ml of 10% acetonitrile (Merck, Germany) and was filtered with a 0.45 μm filter for high performance liquid chromatography analysis.

### Cell Culture and Treatments

Human hepatocytes LO2 cells were purchased from the cell bank of the Chinese Academy of Sciences (Shanghai, China) and cultured in high-glucose Dulbecco’s modified Eagle’s medium (Gibco, United States) supplemented with 10% fetal bovine serum (FBS) at 37°C in 5% CO_2_. After reaching 50% confluence, the cells were exposed to 0.25 mM oleic acid (Aladdin, Shanghai, China) for 24 h to induce insulin resistance in a culture medium containing 2% fetal bovine serum. The oleic acid was dissolved in a culture medium containing 0.5% fatty acid-free bovine serum albumin (BSA) (Nagaoka et al., 2015). And the control cells were administrated with 0.5% fatty acid-free BSA.

### Glucose Consumption Assay

LO2 cells were seeded into 96-well plates in high-glucose Dulbecco’s modified Eagle’s medium (DMEM) supplemented with 10% FBS and divided into control group and model group. When cell confluence reached 50%, the control group was administrated with 0.5% fatty acid-free BSA, whereas 0.25 mM oleic acid was added to the model groups. After incubation for 24 h, the control group were incubated with DMEM containing 10% FBS, whereas the model groups were pretreated with or without 3-methyladenine (3-MA) (Selleckchem, Houston, TX, USA) and rapamycin (RAP) (Selleckchem, Houston, TX, United States) for 1 h, and then treated with KD (100, 200 and 400 μg/ml) or metformin (5 mM) (Sino American Shanghai Squibb Pharmaceutical Co., Ltd) for 24 h. And all groups were incubated with 7.8 × 10^−7^ mol/L insulin for 4 h. And then the glucose concentrations in cell supernatant were determined by the glucose oxidase method according to manufacturers’ instruction (Nanjing Jian cheng, Nanjing, China). And we also analyzed cell viability by 3-(4, 5-dimethylthiazol-2-yl)-2, 5-diphenyltetrazolium bromide (MTT) assay to normalize glucose consumption (Ding et al., 2019).

### Glucose Uptake Assay

LO2 cells were seeded into 24-well plates in DMEM supplemented with 10% FBS and divided into control group and model group. When cell confluence reached 50%, the control group was administrated with 0.5% fatty acid-free BSA, whereas 0.25 mM oleic acid was added to the model groups. After incubation for 24 h, the control group was incubated with DMEM containing 10% FBS, whereas the model groups were treated with KD (100, 200 and 400 μg/ml) or metformin (5 mM) for 24 h. All groups were incubated with 0.5 mM 2-deoxy-2-[(7-nitro-2, 1, 3-benzoxadiazol-4-yl) amino]-D-glucose (2-NBDG) for 30 min and 7.8 × 10^−7^ mol/L insulin for 4 h. Next, cells were washed three times with 1×phosphate buffered saline (PBS) to remove the unabsorbed 2-NBDG. Then, relative fluorescence images were observed under a fluorescence microscope (Nikon, Japan) (Reddy et al., 2019).

### Intracellular Triglyceride and Cholesterol Levels Assay

LO2 cells were seeded into 60 mm dishes in DMEM supplemented with 10% FBS and divided into control group and model groups. When cell confluence reached 50%, the control group was administrated with 0.5% fatty acid-free BSA, whereas 0.25 mM oleic acid was added to the model groups. After incubation for 24 h, the control group was incubated with DMEM containing 10% FBS, whereas the model groups were pretreated with or without 3-MA and RAP for 1 h, and then treated with KD (100, 200 and 400 μg/ml) or metformin (5 mM) for 24 h. Subsequently, intracellular triglyceride, HDL-C and LDL-C levels were analyzed according to the manufacturer’s instructions (Nanjing Jiancheng, Nanjing, China).

### Oil Red O Staining

LO2 cells were seeded into 12-well plates in DMEM supplemented with 10% FBS and divided into control group and model groups. When cell confluence reached 50%, the control group was administrated with 0.5% fatty acid-free BSA, whereas 0.25 mM oleic acid was added to the model groups. After incubation for 24 h, the control group was incubated with DMEM containing 10% FBS, whereas the model groups were pretreated with or without 3-MA and RAP for 1 h, and then treated with KD (100, 200 and 400 μg/ml) or metformin (5 mM) for 24 h. Then cells were fixed with 4% paraformaldehyde for 30 min and stained with oil red O solution for 10 min. Subsequently, cells were washed with 1×PBS, and then observed with a light microscope (Nikon, Japan) (Tan et al., 2019).

To quantify the lipid accumulation, isopropanol was used to dissolve stained lipid droplets and the absorbance was determined at 510 nm by a microplate reader (BioTek) (Romacho et al., 2015).

### Adenovirus Infection

To analyze autophagic flux, LO2 cells were seeded into confocal dishes, and treated with experimental conditions as indicated. Then LO2 cells were infected with adenoviruses expressing mRFP-GFP-tagged LC3 (HANBIO, Shanghai, China). All images were acquired by a LSM 710 confocal laser microscope (Zeiss, Germany). Autophagy flux was evaluated by the number of mRFP^+^/GFP^+^ (yellow) and mRFP^+^/GFP^-^ (red) puncta in cells. Yellow puncta indicated autophagosomes and red puncta represented autolysosomes (Sun et al., 2020). To quantify autophagic flux, GFP-LC3 and mRFP-LC3 punctate dots were counted by Image Pro plus 6.0 software (Media Cybernetics, Silver Spring, MD, United States) (Wang et al., 2019).

### Small Interfering RNA-Mediated Knockdown of Atg7

Small interfering RNA (siRNA) specific to human Atg7 and negative control siRNA were designed and synthesized by *RiboBio* (Guangzhou, China). In brief, cells were transfected with 10 nM Atg7 siRNA and a negative control siRNA using ribo*FECT* ^TM^ CP transfection kit according to manufacturer’s instructions (RiboBio, Guangzhou, China). After 48 h, 0.25 mM oleic acid was added to LO2 cells for 24 h and then KD treatment was performed for 24 h.

### Animals

Male Sprague-Dawley rats (weighing 180–220 g), were purchased from the Medical Laboratory Animal Center of Guangdong Province (Foshan, China). All rats were maintained in a controlled environment of 23–25°C, with a 12 h light/dark cycle, relative humidity of 45–65%, and had free access to regular chow and water. All animal experiments were approved by the Institutional Animal Care and Use Committee of Guangdong Provincial Academy of Chinese Medical Sciences in Guangzhou University of Chinese Medicine (SYXK Guangdong 2013-0094).

### Experimental Design

A total of 72 male rats were randomly divided into two groups: control group (*n* = 12) and high-fat diet group (*n* = 60). The rats in the control group were fed with a normal diet (67% of total calories from carbohydrates, 21% from proteins, and 12% from fat (Soybean Oil), total calories: 3.5 Kcal/g). The rats in the high-fat diet group were fed a high-fat diet (18% of total calories from carbohydrates, 21% from proteins, and 61% from fat (Soybean Oil and Lard), total calories: 5.24 Kcal/g). After six weeks, the body weight, body length, serum LDL-C, HDL-C, glucose and insulin levels in each rat were detected to evaluate whether metabolic syndrome was established. A total of 50 rats in the high-fat diet group were identified as developing metabolic syndrome. Then the rats with metabolic syndrome were randomly assigned into five groups: model group, metformin group (100 mg/kg) and KD groups (0.75, 1.5 and 3.0 g/kg) (*n* = 10). Subsequently, the rats in the model group, metformin group and KD groups were administrated with vehicle, metformin and KD daily for 6 weeks, respectively. However, the rats in the control group were administrated with distilled water instead of drug. 6 weeks later, all rats were euthanized by inhaling isoflurane and the serum, liver, spleen, kidney and abdominal adipose tissues were collected for the mechanism study.

### Oral Glucose Tolerance Test

At the 4th week following treatment, an oral glucose tolerance test (OGTT) was performed after 16 h fasting as described previously (Sacramento et al., 2018). Briefly, fasted rats were orally administered with glucose solution at a dose of 2 g/kg. Blood samples were collected at 0, 30, 60, 90 and 120 min. The blood glucose levels were determined by glucose test kit.

### Insulin Tolerance Test

At the 5th week following treatment, insulin tolerance test (ITT) was performed after 5 h fasting by intraperitoneally injecting insulin at 0.75 U/kg (Rached et al., 2010; Jurowich et al., 2015). Blood samples were collected at 0, 30, 60, 90 and 120 min. The blood glucose levels were determined by glucose test kit.

### Measurement of Body Weight and Obesity

During drug treatment, the body weight and body length (nasal-anal length) of each rat were recorded weekly. Lee’s index was examined to evaluate the magnitude of obesity. The greater Lee’s index means more serious obesity. Lee’s index was calculated according to the following formula (Bernardis and Patterson, 1968; Lei et al., 2007):

$$\text{Lee's index }=\text{body weight }{\left(\text{g}\right)}^{1/3}\times 10^{3}/\text{body length}\left(\text{cm}\right)\text{. }$$

After drug treatment for 6 weeks, all rats were sacrificed, and the liver, spleen, kidney and abdominal adipose tissues were excised and weighed. The coefficient of tissue to body weight was also investigated to estimate the magnitude of obesity according to the following formula (Zhu et al., 2019):

Organ coefficient =organ weight(mg)/body weight (g).

### Measurement of Insulin Sensitivity and Serum Cholesterol, Triglyceride, LDL-C and HDL-C Levels

The blood samples from all rats were collected and centrifuged (4°C, 900 × g, 10 min) to obtain serum. The levels of cholesterol, triglyceride, LDL-C, HDL-C, insulin and glucose in serum were measured according to the manufacturers’ instructions.

The insulin sensitivity index (ISI) and homeostasis model assessment-insulin resistance (HOMA-IR) were calculated according to the following formula (Xiaohong and Wei, 2004):

$$\text{ISI }=\text{ Ln}{\left(\text{glucose}\times \text{insulin}\right)}^{-1}$$

HOMA‐IR = glucose × insulin/22.5

### Histological Examination

After rats were sacrificed, the liver tissues from all rats were cut into small pieces, fixed with 10% buffered formalin overnight and then imbedded in paraffin. After deparaffinization and dehydration, the liver tissue was sectioned at 3 μm thickness and stained with hematoxylin and eosin for histological examination.

### Western Blot Assay of Liver Tissues and LO2 Cells

The total protein of liver tissue and LO2 cells was extracted by radioimmunoprecipitation assay buffer (RIPA) lysis buffer according to the manufacturers’ instructions. After the protein concentration was measured by the BCA protein assay kit (Thermo Fisher, Massachusetts, United States), equal amounts of protein were loaded onto 10% sodium dodecyl sulphate–polyacrylamide gel electrophoresis (SDS-PAGE) and transferred to polyvinylidene difluoride (PVDF) membranes by an electrophoresis system (Bio-Rad Laboratory, California, United States). PVDF membranes were blocked with tris-buffered saline containing 5% non-fat milk for 1.5 h at room temperature. Then the membranes were probed with primary antibodies against AMPK, p-AMPK (Thr172), mTOR, p-mTOR (Ser2448), p62, Atg7, Atg5, Atg12, Atg13, Atg16L1 and LC3-Ⅱ/Ⅰ at 4°C overnight and incubated with horseradish peroxidase-conjugated secondary antibody for 1 h at room temperature. The immunoreactive bands were developed by using enhanced chemiluminescence Western blotting detection reagent.

### Quantitative Real-Time PCR Assay of LO2 Cells

The total RNA was isolated from LO2 cells by RNAiso Plus (Takara, Shiga, Japan) and dissolved in RNAase-free water. Then mRNA was reverse transcribed to cDNA according to the manufacturers’ instructions: 42°C for 15 min, 95°C for 3 min and held at 4°C. Then cDNA was used for real-time polymer chain reaction (PCR) by 7500 Real-Time PCR Detection System (ABI, United States). The real-time PCR conditions: 95°C for 3 min, followed by 40 cycles of 95°C for 5 sec and 60°C for 32 sec. The following primers used in this study were listed in Table 2.

**TABLE 2:** Primers for qPCR of gens used in this study.

Gene	Forward sequence (5’-3’)	Reverse sequence (5’-3’)
AMPK	ACT​ATA​CCA​GGT​GAT​CAG​CAC​TC	TTC​CAT​CTC​TTC​AAC​CCG​TC
Atg13	CAT​GTC​TAC​CAG​GCA​ATT​TGA​G	CCA​GTG​TCC​TCA​CCA​GCA​G
Atg7	CAA​GAC​TGC​AGA​TAA​GAA​GCT​C	GAG​GAG​GAA​CTT​GTT​GAG​GAG
ULK1	TCG​GCA​CCA​TCG​TCT​ACC​A	GGG​ACC​AAC​GTC​TTG​TTC​TTC
mTOR	TGA​ATA​AAG​TTC​TGG​TGC​GAC​A	CGA​TGC​TGG​TAA​ATC​AAA​GGA
ATG5	ATC​TCC​TCA​AAG​AAG​TTT​GTC​CTT​C	GCT​CAG​ATG​TTC​ACT​CAG​CCA​CT
MAP1LC3A	ATG​GTG​AGT​GTG​TCC​ACG​CC	TCA​GAA​GCC​GAA​GGT​TTC​CT
ATG16L1	CTT​TGC​CGT​GAA​TGG​GAT​TT	CCC​AAG​TGA​GGT​ATG​GAA​GGT​C
ATG12	AGT​GAG​AAA​GCC​TTA​GGT​GTT​GAA	CCT​GTA​GCT​GGC​TTC​CTT​AGT​GC

Relative quantification of mRNA level was calculated by using the comparative Ct method with β-actin as the reference gene.

### Statistical Analysis

All data were expressed as mean ± standard deviation (SD). Statistical analysis was performed by SPSS 17.0 statistical software. One-way analysis of variance (ANOVA) and Dunnett’s post hoc test were used for multiple comparisons. *P-values* less than 0.05 were considered as statistically significant.

## Results

### KD-Compound-Target Network Analysis

**FIGURE 1:**
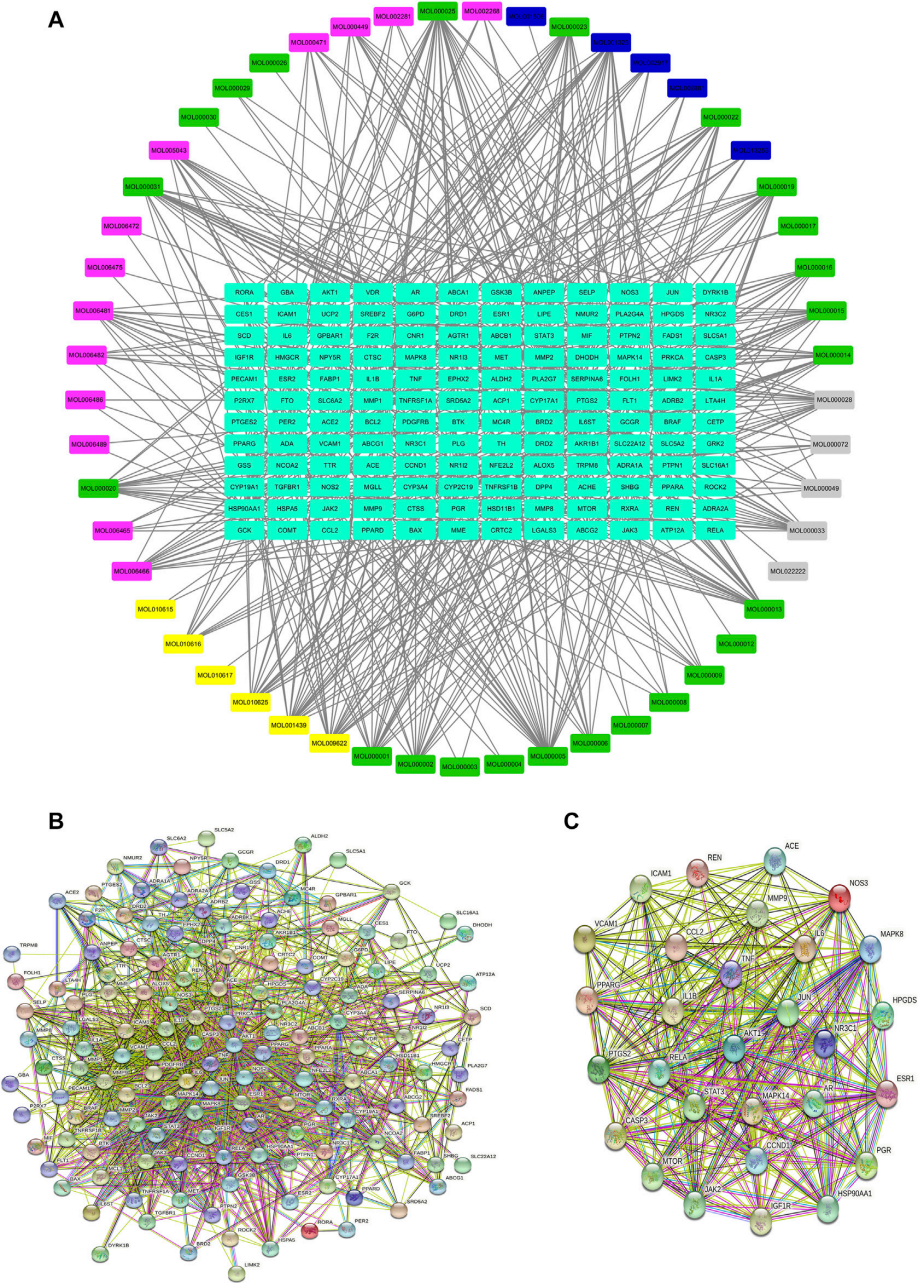
Network pharmacology analysis of KD in the treatment of metabolic syndrome. **(A)** Compound-target network of KD. The network included 52 compounds and 145 proteins, and contained 197 nodes and 554 edges. **(B)** PPI network of the identified targets. **(C)** The hub target of KD in the treatment of metabolic syndrome.

The compound-target network was constructed by using Cytoscape 3.7.2. In the network, the gray nodes represent the chemical compound of *Atractylodes macrocephala* Koidz., the pink nodes represent the chemical compound of *Cassia obtusifolia* L., the yellow nodes represent the chemical compound of *Ecklonia kurome* Okam., the green nodes represent the chemical compound of *Crataegus pinnatifida* Ege., the blue nodes represent the chemical compound of *Citrus medica* L. var. *Sarcodactylis* Swingle, and the light blue nodes represent the target protein. The compound-target network includes 52 compounds and 145 proteins, and also contains 197 nodes and 554 edges (Figure 1, Table 3).

**TABLE 3:** The ingredients in KD-compound-target network.

Drug	Mol ID	Ingredients
*Atractylodes macrocephala* Koidz.	MOL022222	14-acetyl-12-senecioyl-2E,8Z,10E-atractylentriol
	MOL000033	(24S)-24-Propylcholesta-5-ene-3beta-ol
	MOL000049	3β-Acetoxy-atractylone
	MOL000072	8β-Ethoxy atractylenolide Ⅲ
	MOL000028	α-Amyrin
*Citrus medica* L. var. *Sarcodactylis* Swingle	MOL013253	5,2',5'-Trihydroxy-6,7,8-trimethoxyflavone
	MOL002881	Diosmetin
	MOL002917	5,2',6'-Trihydroxy-7,8-dimethoxyflavone
	MOL001506	Supraene
*Cassia obtusifolia* L.	MOL002268	Rhein
	MOL002281	Toralactone
	MOL000449	Stigmasterol
	MOL000471	Aloe-emodin
	MOL006472	Aurantio-obtusin
	MOL006475	1,7-Dihydroxy-2,3,8-trimethoxy-6-methylanthracene-9,10-dione
	MOL006481	Gluco-obtusifolin
	MOL006482	isotoralactone
	MOL006486	Obtusin
	MOL006489	Quinizarin
	MOL005043	Campest-5-en-3beta-ol
	MOL006465	Rubrofusarin-6-beta-gentiobioside
	MOL006466	Rubrofusarin
*Ecklonia kurome* Okam.		
	MOL010615	Saringosterol
	MOL010616	Eckol
	MOL010617	Eicosapentaenoic acid
	MOL010625	24-Methylenecholesterol
	MOL001439	Arachidonic acid
	MOL009622	Fucosterol
*Crataegus pinnatifida* Ege.	MOL000001	1,3,4-trimethyl-3-cyclohexene-1-carboxaldehyde
	MOL000002	1-ethyl-4,8-dimethoxy-beta-carboline
	MOL000003	2-heptanol
	MOL000004	2-methylcyclopentanone
	MOL000005	3,7,11-trimethyldodeca-1,7,10-trien-3-ol-9-one
	MOL000006	3-methyl-1,2-cyclopentanediol
	MOL000007	3-methylhistidin
	MOL000008	4-methylcyclohexanone
	MOL000009	4-p-menthane-1,7,8-triol
	MOL000012	Ascorbic acid
	MOL000013	Caffeic acid dimethyl ether
	MOL000414	Caffeic acid
	MOL000015	Chlorogenin
	MOL000016	Citronellal
	MOL000017	Crataequinones A
	MOL000019	Dimethyl camphorate
	MOL000022	Ethylnotopterol
	MOL000023	Gamma-decanolactone
	MOL000025	Methyl-n-nonylketone
	MOL000026	Methylheptenone
	MOL000029	Proscillaridin a
	MOL000030	Succinic acid
	MOL000031	Suchilactone
	MOL000020	Epicatechin

### Hub Target Identification

The PPI network of KD is shown in Figure 1B, including 145 nodes and 1538 edges. Three topological features of each node in the network were calculated to find the major nodes. Therefore, 29 nodes with an average value of degree ≥30, node betweenness ≥100, and closeness ≥0.4 were considered as major nodes (Figure 1C and Table 4).

**TABLE 4:** Topological features of major nodes.

Major nodes	Degree	Betweenness	Closeness
IL6	89	2278.7522	0.72
AKT1	81	1855.6619	0.6956522
TNF	72	854.31415	0.6635945
PPARG	56	749.75684	0.6180258
PTGS2	62	728.0817	0.62608695
STAT3	55	635.48035	0.6
NOS3	52	570.5138	0.6075949
CCND1	46	566.0214	0.5714286
ACE	43	535.50684	0.56916994
NR3C1	38	509.53333	0.5647059
CASP3	62	500.67346	0.62882096
HPGDS	34	485.54797	0.55172414
AR	39	401.8193	0.55813956
CCL2	50	374.3773	0.5925926
MMP9	52	372.92267	0.5877551
HSP90AA1	44	351.3244	0.5714286
MAPK14	44	351.1595	0.56692916
REN	37	349.07602	0.5475285
IL1B	50	347.06134	0.5925926
JUN	58	342.99088	0.61538464
ESR1	49	333.72092	0.5925926
JAK2	35	299.31857	0.5475285
MAPK8	54	256.67966	0.60504204
RELA	42	255.89087	0.5625
MTOR	42	243.29268	0.5714286
IGF1R	31	207.61827	0.53333336
VCAM1	40	174.15485	0.55598456
PGR	30	138.3102	0.53136533
ICAM1	42	120.25023	0.5625

### GO Enrichment Analysis and KEGG Pathway Analysis

**FIGURE 2:**
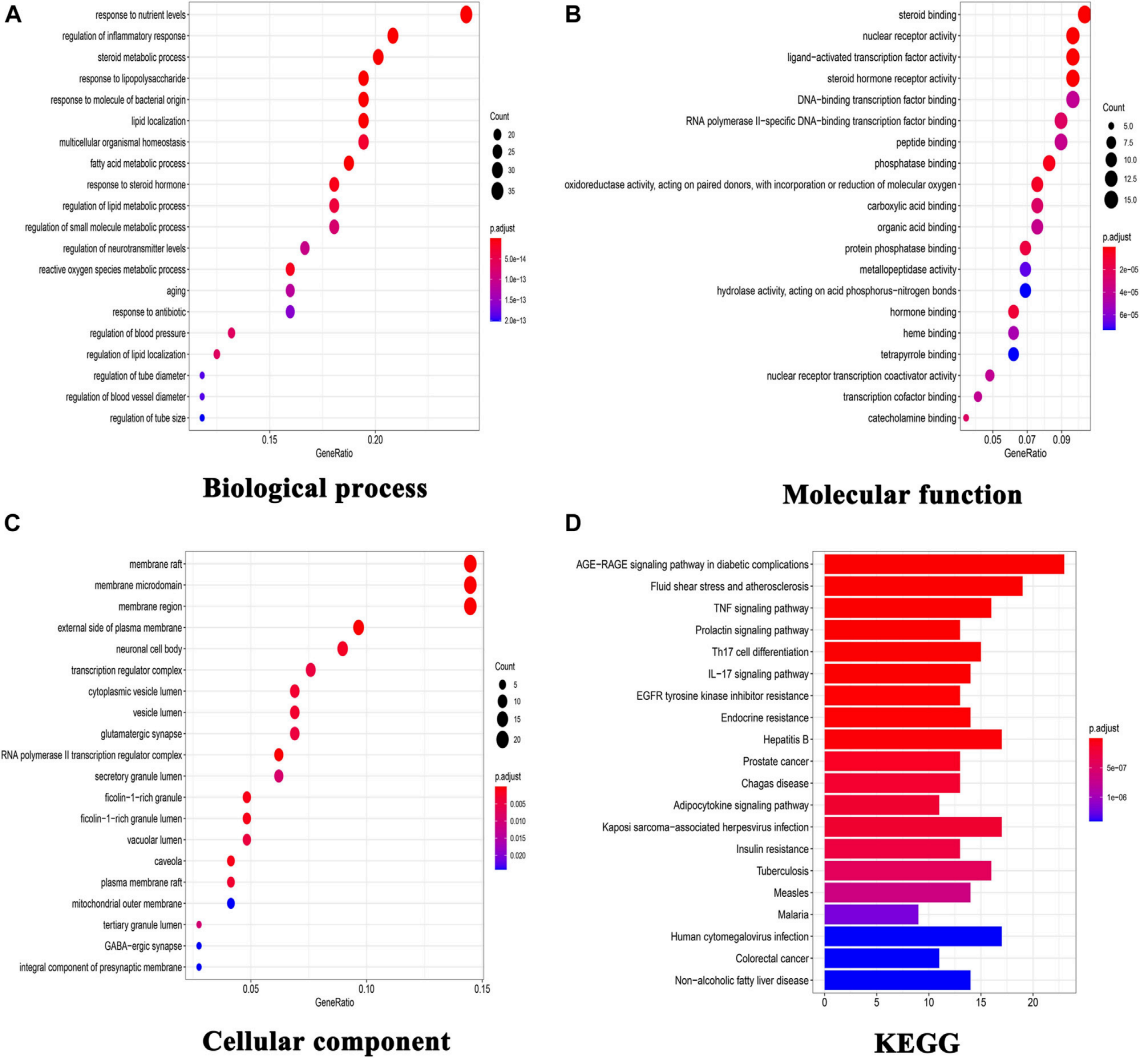
GO functional enrichment analysis and KEGG pathway analysis. **(A)** GO enrichment analysis for biological process; **(B)** GO enrichment analysis for molecular function; **(C)** GO enrichment analysis for cellular component; **(D)** KEGG pathway analysis of these targets.

To explore the underlying mechanism of KD in the treatment of metabolic syndrome, we performed a GO enrichment analysis for biological process, molecular function, and cellular component. As shown in Figure. 2A, the top 10 enrichment results of biological process include response to nutrient levels (GO:0031667), steroid metabolic process (GO:0008202), regulation of inflammatory response (GO:0050727), response to lipopolysaccharide (GO:0032496), response to molecule of bacterial origin (GO:0002237), lipid localization (GO:0010876), fatty acid metabolic process (GO:0006631), response to steroid hormone (GO:0048545), reactive oxygen species metabolic process (GO:0072593) and multicellular organismal homeostasis (GO:0048871). As shown in Figure 2B, the top 10 enrichment results of molecular function include nuclear receptor activity (GO:0004879), ligand-activated transcription factor activity (GO:0098531), steroid hormone receptor activity (GO:0003707), steroid binding (GO:0005496), phosphatase binding (GO:0019902), oxidoreductase activity (GO:0016705), hormone binding (GO:0042562), protein phosphatase binding (GO:0019903), RNA polymerase II-specific DNA-binding transcription factor binding (GO:0061629) and catecholamine binding (GO:1901338). As shown in Figure 2C, the top 10 enrichment results of cellular component include membrane raft (GO:0045121), membrane microdomain (GO:0098857), membrane region (GO:0098589), external side of plasma membrane (GO:0009897), RNA polymerase II transcription regulator complex (GO:0090575), caveola (GO:0005901), ficolin-1-rich granule (GO:0101002), ficolin-1-rich granule lumen (GO:1904813), neuronal cell body (GO:0043025) and cytoplasmic vesicle lumen (GO:0060205).

140 target-enriched KEGG pathways were analyzed in our study. And the top 20 pathways are demonstrated in Figure 2D. Some pathways are closely related to metabolic syndrome, including advanced glycation end products (AGE)-receptor of AGE (RAGE) signaling pathway in diabetic complications (hsa04933), fluid shear stress and atherosclerosis (hsa05418), TNF signaling pathway (hsa04668), adipocytokine signaling pathway (hsa04920), insulin resistance (hsa04931) and non-alcoholic fatty liver disease (hsa04932).

### HPLC Analysis of KD

**FIGURE 3:**
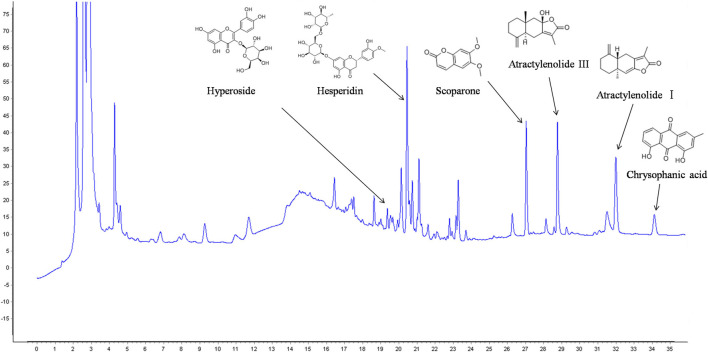
HPLC analysis of main components in KD.

Chromatogram of KD is depicted in Figure 3. Hyperoside, hesperidin, scoparone, atractylenolide Ⅲ, atractylenolide Ⅰ and chrysophanic acid are identified in KD by comparing the retention time and UV spectra of reference standards. Hyperoside is derived from *Crataegi* Fructus. Hesperidin and scoparone are derived from *Fructus Citri* Sarcodactylis. Atractylenolide Ⅲ and atractylenolide Ⅰ are derived from *Rhizoma Atractylodis* Macrocephalae, and chrysophanic acid is derived from *Semen* Cassiae.

### KD Enhances Insulin Sensitivity in Insulin-Resistant LO2 Cells

**FIGURE 4:**
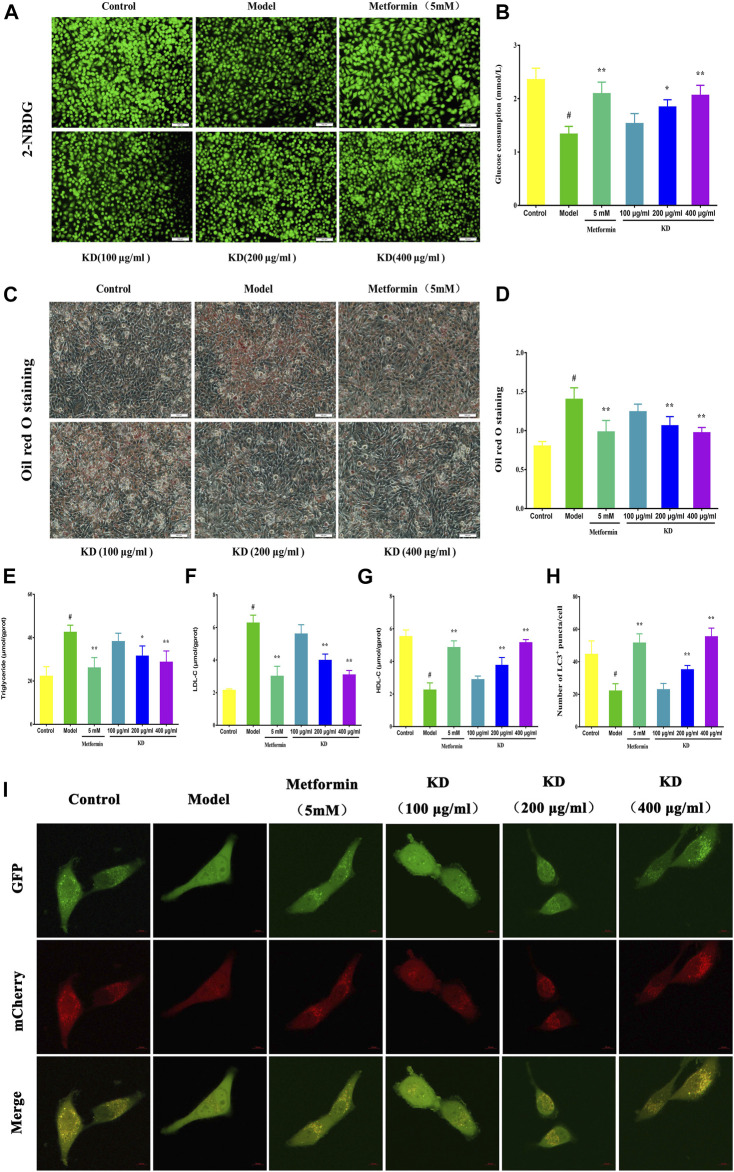
KD activates hepatic autophagy to improve the glucose and lipid metabolism in LO2 cells. LO2 cells were incubated in DMEM with 0.5% fatty acid-free BSA or oleic acid (0.25 mM) in the presence of KD (100, 200 and 400 μg/ml) or metformin (5 mM) for 24 h. 2-NBDG and glucose oxidase and peroxidase assay were used to analyze the glucose uptake **(A)** and consumption **(B)** of oleic acid-induced LO2 cells. Oil red O staining was performed to evaluate the lipid accumulation of insulin-resistant cells **(C,D)**. Intercellular triglyceride **(E)**, LDL-C **(F)** and HDL-C **(G)** were also determined to assess the lipid metabolism of insulin-resistant cells. **(I)** Then LO2 cells were infected with adenoviruses expressing mRFP-GFP-tagged LC3. All images were acquired by a confocal laser microscope. **(H)** Autophagy flux was assessed by the number of mRFP^+^/GFP^+^ (yellow) and mRFP^+^/GFP^-^ (red) puncta in cells. Yellow puncta represents autophagosomes and red puncta represents autolysosomes. Data were presented as mean ± SD (*n* = 3 in each group). ^#^ *P* < 0.01 compared with the control group, *P < 0.05, **P < 0.01 compared with the model group.

Impaired glucose tolerance is a key characteristic of insulin resistance. Therefore, the glucose uptake and consumption of LO2 cells were determined in our study. Compared with the control group, glucose uptake was remarkably inhibited in insulin-resistant cells, which were manifested by significantly reduced fluorescence density and area (Figure 4A). However, we found that KD treatment concentration-dependently enhanced the glucose uptake in oleic acid-induced cells (Figure 4A). As shown in Figure 4B, the glucose consumption of insulin-resistant LO2 cells is also lower than LO2 cells without insulin resistance. As we expected, KD at doses of 200 and 400 μg/ml significantly enhanced the glucose consumption of insulin-resistant LO2 cells and showed a dose-dependent manner (Figure 4B). Taken together, these results suggest that KD possesses the ability to enhance insulin sensitivity in insulin-resistant LO2 cells.

### KD Prevents Lipid Accumulation in Insulin-Resistant Cells

Growing evidence has shown that lipid accumulation in hepatocytes is the principal risk of insulin resistance. Therefore, we analyzed intercellular triglyceride, LDL-C and HDL-C contents to evaluate the inhibitory effect of KD on lipid accumulation in insulin-resistant LO2 cells. The results of oil red o staining showed that KD at doses of 200 and 400 μg/ml dramatically prevented the lipid accumulation in insulin-resistant cells (Figures 4C–D). Intercellular triglyceride (Figure 4E) and LDL-C (Figure 4F) contents were also inhibited by KD, whereas HDL-C (Figure 4G) expression was significantly increased by KD. In a word, KD can prevent the lipid accumulation in hepatocytes, which may contribute to the treatment of insulin resistance.

### KD Activates Autophagy to Improve Insulin Resistance in Hepatocytes

**FIGURE 5:**
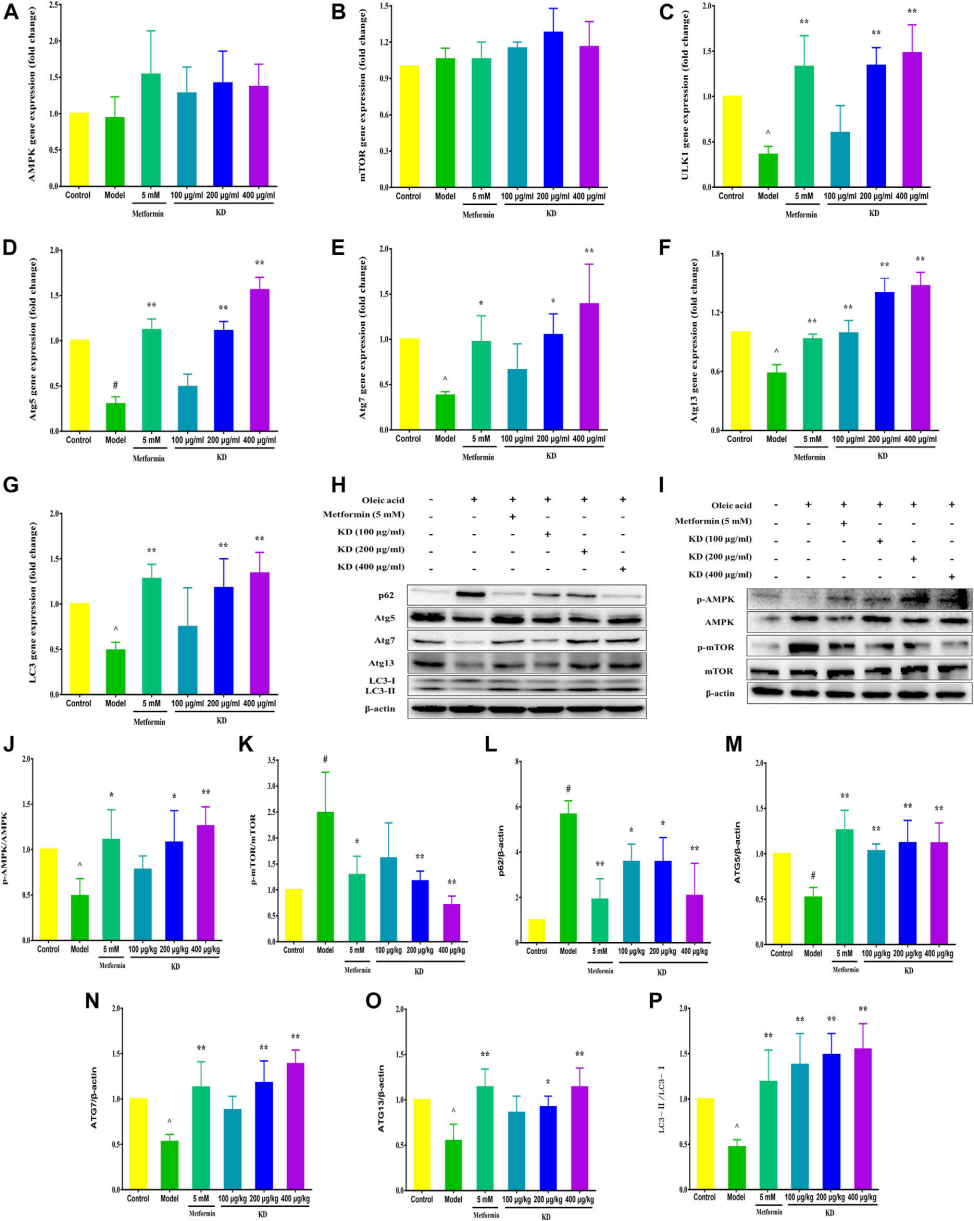
KD enhances the expressions of autophagy-related proteins and genes in insulin-resistant cells. LO2 cells were incubated in DMEM with 0.5% fatty acid-free BSA or oleic acid (0.25 mM) in the presence of KD (100, 200 and 400 μg/ml) or metformin (5 mM) for 24 h. The expressions of AMPK **(A)**, mTOR **(B)**, ULK1 **(C)**, Atg5 **(D)**, Atg7 **(E)**, Atg13 **(F)** and LC3 **(G)** genes in hepatocytes were analyzed. **(H–P)** Western blot analysis for AMPK, p-AMPK (Thr172) **(J)**, mTOR, p-mTOR (Ser2448) **(K)**, p62 **(L)**, Atg5 **(M)**, Atg7 **(N)**, Atg13 **(O)** and LC3-Ⅱ/Ⅰ **(P)**. Data were presented as mean ± SD (*n* = 3 in each group). #*P* < 0.01 compared with the control group, *P < 0.05, **P < 0.01 compared with the model group.

Growing evidence shows that impaired autophagy is responsible for hepatic insulin resistance (Shiwei et al., 2014). To elucidate the underlying mechanism of autophagy in the treatment of insulin resistance by KD, we determined the autophagic flux and related protein expressions of hepatocytes by adenoviruses expressing mRFP-GFP-tagged LC3 and western blot assay. Consistent with previous studies, autolysosomes (red puncta) were significantly increased by KD, indicating that KD could effectively enhance the autophagy flux of insulin-resistant LO2 cells (Figures 4H,I). We also observed that KD dramatically increased the gene expressions of ULK1 (Figure 5C), Atg5 (Figure 5D), Atg7 (Figure 5E), Atg13 (Figure 5F) and LC3 (Figure 5G) in hepatocytes**.** The western blot results also showed that KD observably increased the protein expressions of p-AMPK (Figure 5J), Atg5 (Figure 5M), Atg7 (Figure 5N), Atg13 (Figure 5O) and LC3-Ⅱ/Ⅰ (Figure 5P), and inhibited p-mTOR (Figure 5K) and p62 (Figure 5L) expressions in hepatocytes (Figures 5H–I). Taken together, we speculate that autophagy may play an important role in the treatment of insulin resistance by KD.

### 3-Methyladenine Reverses the Effect of KD on Insulin Resistance

**FIGURE 6:**
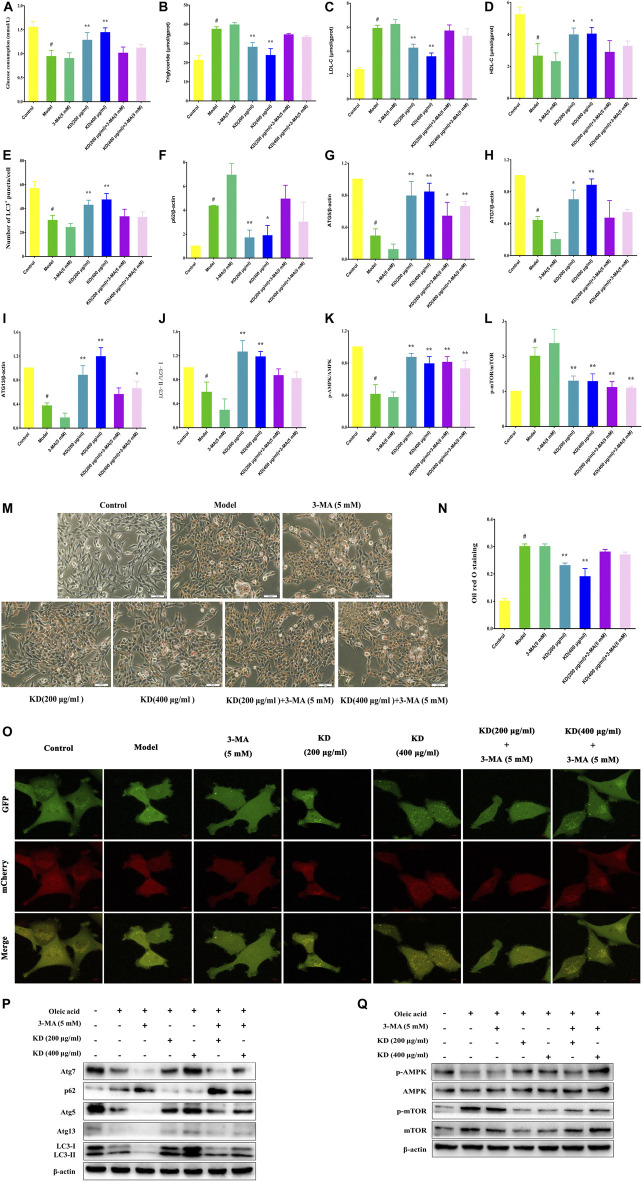
3-MA weakens the therapeutic effect of KD on insulin resistance in LO2 cells. LO2 cells were incubated in DMEM with 0.5% fatty acid-free BSA or oleic acid (0.25 mM) for 24 h, and then were pretreated with 3-MA (5 mM) for 1 h and were treated with or without KD (200 and 400 μg/ml) for 24 h. The glucose consumption of hepatocytes **(A)**; The intercellular triglyceride **(B)**, LDL-C **(C)** and HDL-C **(D)** contents of oleic acid-induced hepatocytes; The autophagy flux of hepatocyte **(E and O)**; the oil red O staining of oleic acid-induced hepatocytes **(M,N)**; Western blot analysis for p62 **(F)**, Atg5 **(G)**, Atg7 **(H)**, Atg13 **(I)**, LC3-Ⅱ/Ⅰ **(J)**, AMPK, p-AMPK (Thr172) **(K)**, mTOR and p-mTOR (Ser2448) **(L) (P,Q)**. Data were presented as mean ± SD (*n* = 3 in each group). ^#^ *P* < 0.01 compared with the control group, *P < 0.05, **P < 0.01 compared with the model group.

To confirm the role of autophagy in the treatment of insulin resistance by KD, autophagy inhibitor 3-MA was used to verify the molecular mechanism. As exhibited in Figure 6A, 3-MA significantly inhibits KD-promoted glucose consumption in insulin-resistant hepatocytes. And 3-MA also increased intercellular triglyceride (Figure 6B) and LDL-C (Figure 6C), inhibited intercellular HDL-C (Figure 6D) and deteriorated the lipid accumulation in hepatocytes (Figures 6M,N) in the presence of KD. Mechanistically, 3-MA remarkably prevented KD-promoted autophagy flux (Figures 6E,O), and inhibited the protein expressions of Atg5 (Figure 6G), Atg7 (Figure 6H), Atg13 (Figure 6I) and LC3-Ⅱ/Ⅰ (Figure 6J), and increased p62 protein expression (Figure 6F) (Figures 6P,Q). However, the protein expressions of p-AMPK and p-mTOR were not affected by 3-MA (Figures 6K,L,P,Q). These results confirm the key role of autophagy in the therapeutic effect of KD on insulin resistance.

### Rapamycin Enhances the Therapeutic Effect of KD on Insulin Resistance

**FIGURE 7:**
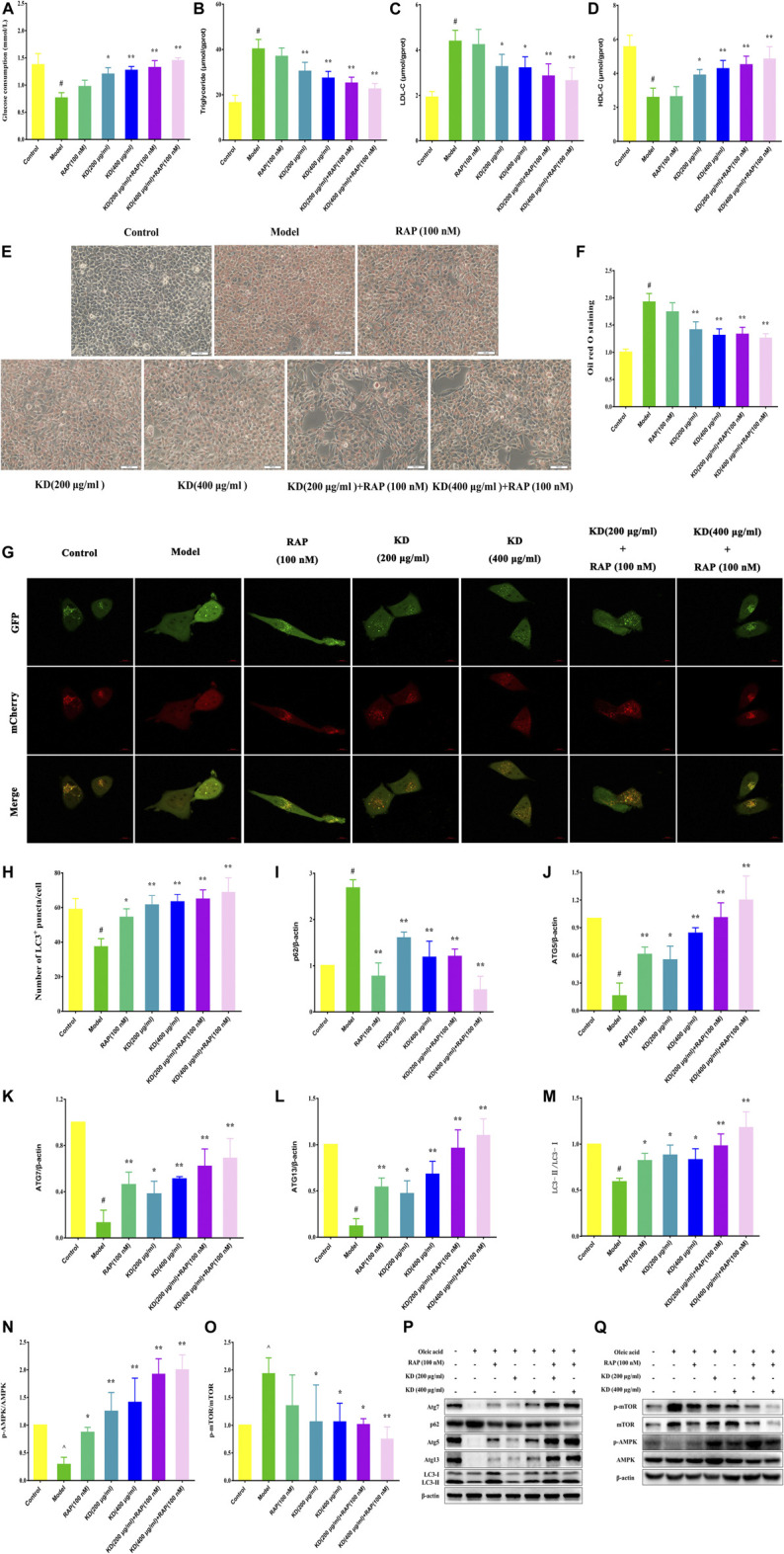
Rapamycin strengthens the therapeutic effect of KD on insulin resistance in hepatocytes. LO2 cells were incubated in DMEM with 0.5% fatty acid-free BSA or oleic acid (0.25 mM) for 24 h, and then were pretreated with RAP (100 nM) for 1 h and were treated with or without KD (200 and 400 μg/ml) for 24 h. **(A)** The glucose consumption of hepatocytes; **(B**–**D)** The intercellular triglyceride, LDL-C and HDL-C contents of oleic acid-induced hepatocytes; **(E**–**F)** The oil red O staining of oleic acid-induced hepatocytes; **(G**–**H)** The autophagy flux of hepatocytes; **(I**–**Q)** Western blot analysis for p62 **(I)**, Atg5 **(J)**, Atg7 **(K)**, Atg13 **(L)**, LC3-Ⅱ/Ⅰ **(M)**, AMPK, p-AMPK (Thr172) **(N)**, mTOR and p-mTOR (Ser2448) **(O)**. Data were presented as mean ± SD (*n* = 3 in each group). ^#^ *P* < 0.01 compared with the control group, *P < 0.05, **P < 0.01 compared with the model group.

Autophagy activator rapamycin (RAP) was also used to verify the therapeutic effect of KD on insulin resistance in our study. As demonstrated in Figure 7A, RAP (100 nM) further heightens KD-promoted glucose consumption in insulin-resistant hepatocytes. And RAP also further inhibited intercellular triglyceride (Figure 7B) and LDL-C (Figure 7C) expressions, promoted intercellular HDL-C content (Figure 7D), and attenuated the lipid accumulation in hepatocytes (Figures 7E–F) in the presence of KD. Mechanistically, rapamycin could cooperate with KD to further enhance autophagic activation (Figures 7G,H). The western blot analysis also revealed that RAP could further promote the protein expressions of Atg5 (Figure 7J), Atg7 (Figure 7k), Atg13 (Figure 7L), LC3-Ⅱ/Ⅰ (Figure 7M) and p-AMPK (Figure 7N), and inhibit p-mTOR (Figure 7O) and p62 (Figure 7I) expressions (Figures 7I–Q). These results indicate that autophagy should be the critical mechanism of KD in the treatment of insulin resistance.

### Atg7 Deficiency Impairs the Therapeutic Effect of KD on Insulin Resistance

**FIGURE 8:**
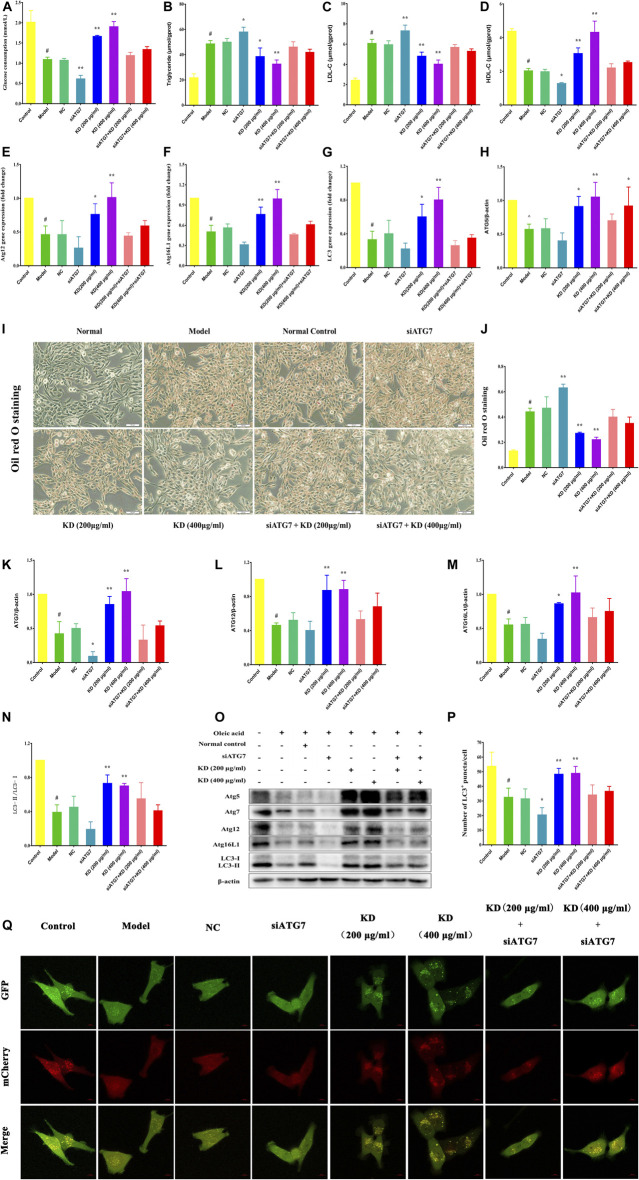
Atg7 deficiency impairs the therapeutic effect of KD on insulin resistance in hepatocytes. LO2 cells were transfected with 10 nM Atg7 siRNA and a negative control siRNA. After 48 h, 0.25 mM oleic acid was added to LO2 cells for 24 h and then KD treatment was performed for 24 h. **(A)** The glucose consumption of hepatocytes; **(B**–**D)** The intercellular triglyceride, LDL-C and HDL-C contents of oleic acid-induced hepatocytes; **(E)** The expression of Atg12 gene; **(F)** The expression of Atg16L1 gene; **(G)** The expression of LC3 gene; **(I,J)** The oil red O staining of oleic acid-induced hepatocytes; **(O)** Western blot analysis for Atg5 **(H)**, Atg7 **(K)**, Atg12 **(L)**, Atg16L1 **(M)** and LC3-Ⅱ/Ⅰ **(N)**; **(P,Q)** The autophagy flux of hepatocytes. Data were presented as mean ± SD (*n* = 3 in each group). ^#^ *P* < 0.01 compared with the control group, *P < 0.05, **P < 0.01 compared with the model group.

To further elucidate the vital role of autophagy in the therapeutic effect of KD on insulin resistance, we down-regulated the autophagy gene Atg7 in hepatocytes using small interfering RNA (siRNA). As shown in Figure 8, Atg7 siRNA dramatically inhibits KD-promoted glucose consumption in insulin-resistant hepatocytes (Figure 8A), increases intercellular triglyceride (Figure 8B) and LDL-C levels (Figure 8C), and inhibits intercellular HDL-C (Figure 8D) contents and deteriorates the lipid accumulation in hepatocytes in the presence of KD (Figures 8I,J). Mechanistically, Atg7 siRNA remarkably prevented KD-promoted autophagy flux (Figures 8P,Q), and inhibited the gene expressions of Atg12 (Figure 8E), Atg16L1 (Figure 8F) and LC3 (Figure 8G), and suppressed the protein expressions of Atg5 (Figure 8H), Atg7 (Figure 8K), Atg12 (Figure 8L), Atg16L1 (Figure 8M) and LC3-Ⅱ/Ⅰ (Figures 8N,O). In conclusion, these results further confirm the key role of autophagy in the therapeutic effect of KD on insulin resistance.

### KD Inhibits Obesity in High-Fat Diet-Fed Rats

**FIGURE 9:**
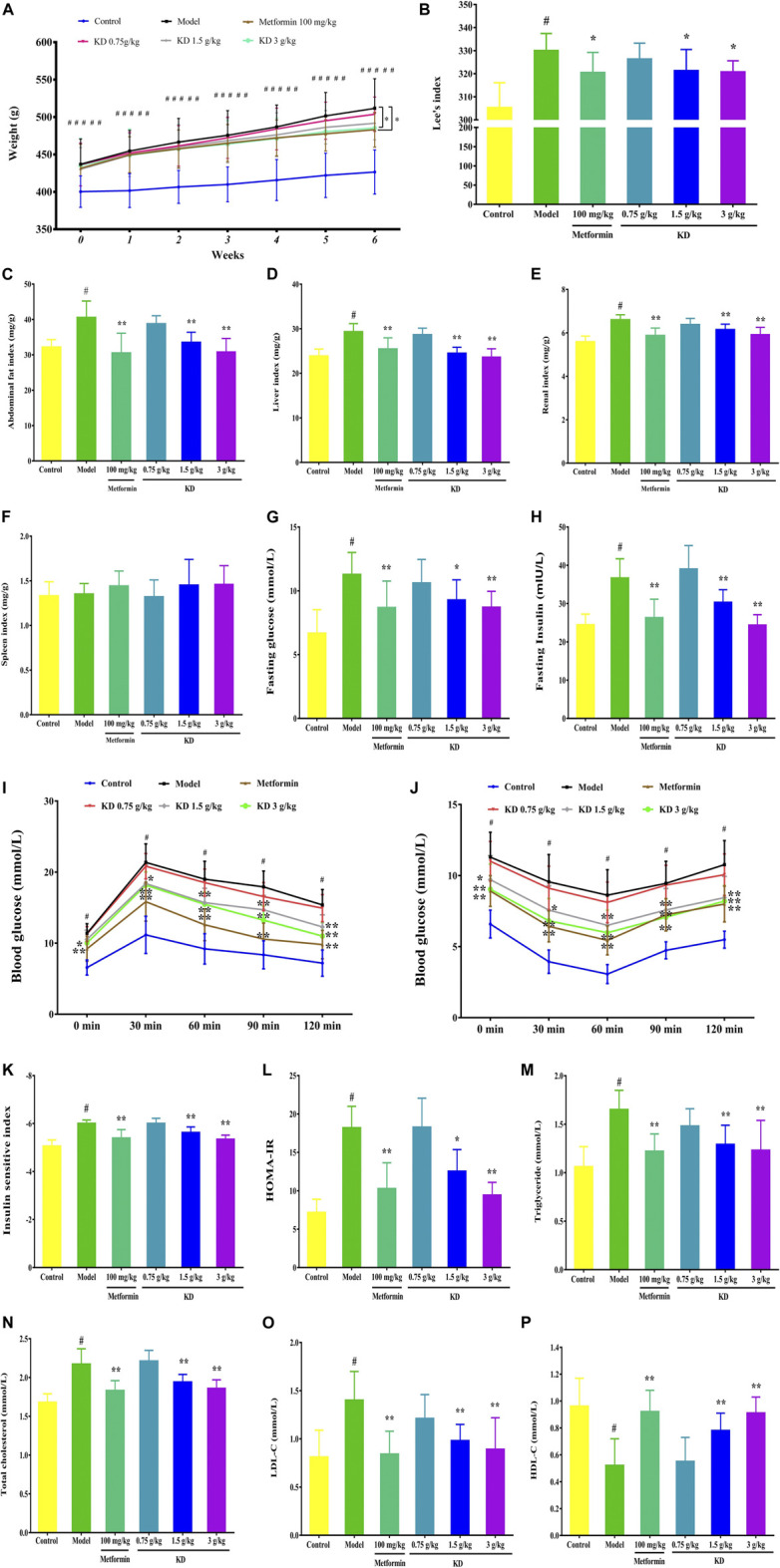
KD improves the obesity and insulin sensitivity of high-fat diet-fed rats. **(A)** Effect of KD on the body weights of rats. **(B)** Effect of KD on the Lee’s indexes of rats. **(C)** The abdominal fat indexes of rats. **(D)** The liver indexes of rats. **(E)** The renal indexes of rats. **(F)** The spleen indexes of rats. **(G)** Fasting glucose in blood. **(H)** Fasting insulin in blood. **(I)** Effect of KD on the oral glucose tolerance in rats. **(J)** Effect of KD on the insulin tolerance in rats. **(K)** Insulin sensitivity index. **(L)** HOMA-IR. **(M)** The serum triglyceride levels of rats. **(N)** The serum cholesterol levels of rats. **(O)** The serum LDL-C levels of rats. **(P)** The serum HDL-C levels of rats. Data were presented as mean ± SD (*n* = 7–10 in each group). ^#^ *P* < 0.01 compared with the control group, *P < 0.05, **P < 0.01 compared with the model group.

Obesity is a prominent characteristic of metabolic syndrome. In high-fat diet-fed rats, excessive fat accumulates in the abdomen, kidney, spleen and liver tissues resulting in an increased weight of these tissues. Therefore, we attempted to examine the body weight, Lee’s index, abdominal fat indexes, renal indexes, spleen indexes and liver indexes of high-fat diet-fed rats to assess the therapeutic effect of KD on obesity. As shown in Figure 9A, at the beginning of treatment, the body weight of rats in the control group is significantly lower than that of the model group, metformin group and KD groups, suggesting that a high-fat diet successfully induces obesity in rats in our study. After 6 weeks of pharmacotherapy, the body weight of rats in the metformin group and the KD group (3.0 g/kg) were obviously attenuated compared with that in the model group (Figure 9A). Furthermore, we observed that KD at doses of 1.5 g/kg and 3.0 g/kg could markedly attenuate the Lee’s indexes of high-fat diet-fed rats (Figure 9B).

We also found that KD (1.5 and 3.0 g/kg) significantly suppressed the abdominal fat indexes (Figure 9C), liver indexes (Figure 9D), and renal indexes (Figure 9E) of high-fat diet-fed rats. However, no significant difference was observed in the spleen indexes (Figure 9F) between each group. In a word, these results suggest that KD can effectively suppress the obesity of high-fat diet-fed rats.

### KD Enhances Insulin Sensitivity in High-Fat Diet-Fed Rats

Insulin resistance is the pathobiologic basis of metabolic syndrome. There is a growing consensus that enhancing insulin sensitivity is an important therapeutic strategy for metabolic syndrome. Therefore, fasting insulin and glucose, OGTT and ITT were examined to investigate the effect of KD on the insulin sensitivity of high-fat diet-fed rats. As shown in Figures 9G–H, the fasting glucose and insulin are prominently increased in the model group (Figures 9G–H). After 6 weeks of pharmacotherapy, the fasting glucose and insulin of rats in KD groups (1.5 g/kg and 3.0 g/kg) were significantly inhibited and showed a dose-dependent manner (Figures 9G–H). In OGTT, the impaired glucose tolerance of high-fat diet-fed rats in the model group was recorded (Figure 9I). However, compared with the model group, KD obviously reduced the blood glucose levels of high-fat diet-fed rats, indicating improved glucose tolerance (Figure 9I). In addition, KD dramatically improved the glucose utilization capacity, indicating that improved insulin tolerance when compared with the model group (Figure 9J). Furthermore, we investigated the insulin sensitivity index and HOMA-IR of high-fat diet-fed rats to evaluate the effect of KD on insulin sensitivity. Compared with the control group, the insulin sensitivity index (Figure 9K) of rats in the model group was prominently inhibited and the increased HOMA-IR (Figure 9L) was also found in the model group. These results indicated that a high-fat diet successfully induced insulin resistance in rats in our study. Interestingly, after 6 weeks of pharmacotherapy, KD effectively enhanced the insulin sensitivity index (Figure 9K) and inhibited the HOMA-IR (Figure 9L) in high-fat diet-fed rats. Taken together, these findings indicate that KD exerts an enhancing effect on insulin sensitivity in high-fat diet-fed rats, which might be beneficial to the treatment of metabolic syndrome.

### KD Inhibits Hyperlipidemia in High-Fat Diet-Fed Rats

Increasing evidence shows that increased serum cholesterol, triglyceride and LDL-C content and reduced serum HDL-C level are found in metabolic syndrome patients. Considering that KD can significantly attenuate the obesity of high-fat diet-fed rats, we further investigated the effect of KD on the serum cholesterol, triglyceride, LDL-C and HDL-C concentrations of high-fat diet-fed rats. As depicted in Figure 9, compared with the control group, the serum triglyceride (Figure 9M), cholesterol (Figure 9N) and LDL-C levels (Figure 9O) of the rats in the model group are remarkably elevated. Conversely, KD (1.5 g/kg and 3.0 g/kg) and metformin (100 mg/kg) had significant inhibitory activity on the overexpression of serum triglyceride (Figure 9M), cholesterol (Figure 9N) and LDL-C levels (Figure 9O). Meanwhile, KD (1.5 g/kg and 3.0 g/kg) also enhanced the expression of serum HDL-C (Figure 9P) compared with the model group. These results indicate that KD effectively inhibits hyperlipidemia in high-fat diet-fed rats.

### KD Prevents Hepatic Steatosis in High-Fat Diet-Fed Rats

**FIGURE 10:**
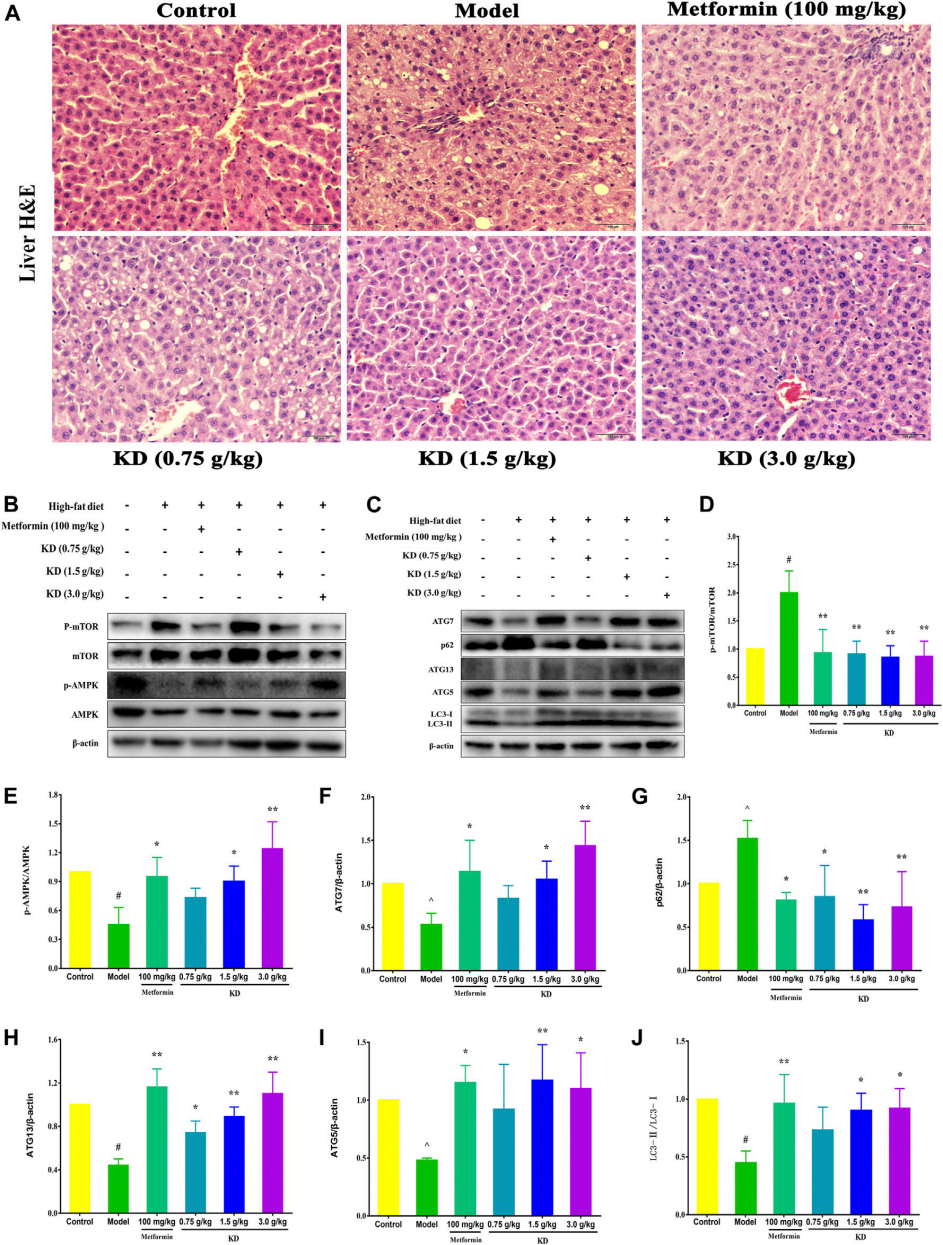
KD activates autophagy to prevent hepatic steatosis in high-fat diet-fed rats. SD rats were fed a normal chow diet or a high-fat diet for 6 weeks, then administrated with metformin (100 mg/kg) or KD (0.75 g/kg, 1.5 g/kg and 3.0 g/kg), and sacrificed 6 weeks later. **(A)** Liver sections were stained with H&E, which shows tissue composition, lipid droplets, and hepatocyte swelling in the livers of high-fat diet-fed rats. **(B**–**J)** Western blot analysis for mTOR, p-mTOR (Ser2448) **(D)**, AMPK, p-AMPK (Thr172) **(E)**, Atg7 **(F)**, p62 **(G)**, Atg13 **(H)**, Atg5 **(I)** and LC3-Ⅱ/Ⅰ **(J)**. Data were presented as mean ± SD (*n* = 3 in each group).

As shown in Figure 10A, hepatocytes with central nuclei radiate from the central vein lined by flat endothelial cells in the control group. Pathological evaluation of liver tissues from the model group manifested many features of hepatic steatosis, including intumescent hepatocytes with eccentric nuclei, plentiful lipid droplets and fatty degenerations with granular cytoplasm. Consistent with the inhibitory effect of hyperlipidemia, KD at doses of 1.5 and 3.0 g/kg significantly attenuated the accumulation of lipid droplets in the liver tissues. However, KD at a dose of 0.75 g/kg could not effectively inhibit the hepatic steatosis of high-fat diet-fed rats.

### KD Activates AMPK/mTOR Signaling Pathway to Trigger Autophagy

To gain insight into the molecular mechanism of KD in the treatment of metabolic syndrome, we further examined the expression of AMPK/mTOR signaling pathway in liver tissues of high-fat diet-fed rats. Compared with the control group, the protein expressions of p-AMPK (E), Atg7 (F), Atg13 (H), Atg5 (I) and LC3-Ⅱ/Ⅰ (J) were remarkably inhibited in the model group, and p-mTOR (D) and p62 (G) protein expressions were increased (Figures 10B–J). However, metformin (100 mg/kg) or KD (1.5 and 3.0 g/kg) efficiently increased the protein expressions of p-AMPK **(**E), Atg7 (F), Atg13 (H), Atg5 (I) and LC3-Ⅱ/Ⅰ (J), and inhibited p-mTOR (D) and p62 (G) protein expressions. Nevertheless, there were no statistically significant differences in the protein expressions of total AMPK and mTOR among all groups. These results suggest that autophagy may be the critical molecular mechanism of KD in the treatment of metabolic syndrome.

## Discussion

Metabolic syndrome is described as a cluster of metabolic alterations including insulin resistance, hyperglycemia, hyperlipidemia, hypertension and obesity. Nowadays, it is generally believed that metabolic syndrome is a risk factor for type 2 diabetes mellitus, cardiovascular disease and cancer. Now, about 20% of adults suffer from metabolic syndrome worldwide (Kuo et al., 2019). Encouragingly, more and more medicinal and edible herbs have shown therapeutic effects on metabolic syndrome. Kun-Dan (KD), comprising *Atractylodes macrocephala* Koidz., *Crataegus pinnatifida* Ege., *Citrus medica* L. var. *Sarcodactylis* Swingle, *Cassia obtusifolia* L. and *Ecklonia kurome* Okam., has been used to treat patients with metabolic syndrome for more than ten years. However, the underlying mechanism of KD in the treatment of metabolic syndrome remains unclear. More and more evidence verifies that impaired autophagy plays a critical risk role in the pathogenesis of metabolic syndrome (Hyejin et al., 2018; Kosacka et al., 2018). Therefore, in this study, we established a rat model of metabolic syndrome and insulin-resistant LO2 cells to explore the role of autophagy in the treatment of metabolic syndrome by KD.

Traditional Chinese medicine is a valuable therapeutic strategy and drug resource for the treatment of metabolic disorders including metabolic syndrome, type 2 diabetes and insulin resistance (Li et al., 2014; Pan and Kong, 2018; He et al., 2019). However, it is a challenge to explore the active ingredients of traditional Chinese medicine and their potential molecular mechanisms. Recently, traditional Chinese medicine network pharmacology has been put forward, which will provide a new research paradigm for the transformation of traditional Chinese medicine empirical medicine to an evidence-based medical system, which will accelerate the discovery of traditional Chinese medicine (Li et al., 2010; Li, 2011; Li and Zhang, 2013).

First, we take advantage of network pharmacology to explore the molecule mechanism of KD in the treatment of metabolic syndrome. In our research, we obtained 52 chemical ingredients for KD and predicted 145 potential targets. The KEGG analysis indicated that KD treated metabolic syndrome by regulating certain signaling pathways including AGE-RAGE signaling pathway in diabetic complications (hsa04933), TNF signaling pathway (hsa04668), adipocytokine signaling pathway (hsa04920), insulin resistance (hsa04931) and non-alcoholic fatty liver disease (hsa04932). In a word, results from network pharmacology suggest that insulin resistance may be the key molecule mechanism of KD in the treatment of metabolic syndrome. Accordingly, considering the important role of insulin resistance in the pathogenesis of metabolic syndrome, we wanted to confirm the role of insulin resistance in the treatment of metabolic syndrome by KD.

In general, insulin stimulates glucose disposal in adipose and muscle tissues, and suppresses hepatic glycogenolysis and gluconeogenesis to maintain glucose homeostasis. In the case of insulin resistance, normal circulating levels of insulin are inadequate to elicit normal insulin responses in adipose, muscle and liver tissues, resulting in hyperglycemia and hyperlipidemia, ultimately metabolic syndrome (Reza and Khosrow, 2009).

In the insulin-resistant cell model, we found that KD effectively enhanced insulin sensitivity and inhibited lipid accumulation in insulin-resistant LO2 cells. Mechanistically, we observed that KD could restore AMPK/mTOR-mediated autophagy to improve the glucose and lipid metabolism of insulin-resistant LO2 cells. Moreover, autophagy activator RAP, inhibitor 3-MA and Atg7 siRNA were used to verify the role of AMPK/mTOR-mediated autophagy in the treatment of metabolic syndrome by KD. In our study, we observed that RAP could enhance the therapeutic effect of KD on the glucose and lipid metabolism of insulin-resistant LO2 cells. However, 3-MA could inhibit the autophagy of insulin-resistant LO2 cells to overthrow the therapeutic effect of KD on glucose and lipid metabolism in insulin-resistant LO2 cells. Moreover, when we knocked out the Atg7 gene in LO2 cells, the therapeutic effect of KD on the glucose and lipid metabolism in insulin-resistant LO2 cells was attenuated. Therefore, we speculate that AMPK/mTOR-mediated autophagy plays a key role in the treatment of metabolic syndrome by KD.

Our study also revealed that KD effectively decreased the body weight, liver indexes, renal indexes and abdominal fat indexes of high-fat diet-fed rats, suggesting that KD could significantly inhibit the obesity of high-fat diet-fed rats. And reduced serum glucose, insulin, cholesterol, triglyceride and LDL-C levels and enhanced serum HDL-C level were observed in high-fat diet-fed rats treated by KD. Moreover, KD also enhanced ISI and inhibited HOMA-IR of high-fat diet-fed rats. Mechanistically, we confirm that KD can activate AMPK/mTOR-mediated autophagy to treat metabolic syndrome in high-fat diet-fed rats.

Recent research indicates that a high-fat diet is a critical risk factor for metabolic syndrome. The rat model of metabolic syndrome is characterized by obesity, hyperinsulinemia and hyperlipidemia, which are the common clinical features of patients with metabolic syndrome. Therefore, a rat model of high-fat diet-induced metabolic syndrome is extensively considered as an ideal and reproducible pharmacological research model (Ruedaclausen et al., 2011; Nascimento et al., 2013). So, we used a rat model of high-fat diet-induced metabolic syndrome to probe into the therapeutic effect of KD on metabolic syndrome in our study.

Obesity is a very important feature of metabolic syndrome (Gepstein and Weiss, 2019). And overexpression of serum cholesterol, triglyceride and LDL-C is also important symptoms in patients with metabolic syndrome (Basu, 2019). In our study, after 12 weeks of high-fat diet, the obesity of rats in the model group increased significantly. Interestingly, after 6 weeks of pharmacotherapy, KD could obviously inhibit the obesity of high-fat diet-fed rats, which were manifested by decreased body weight, Lee’s indexes and abdominal fat, renal and liver indexes. We also found that the serum cholesterol, triglyceride and LDL-C levels in high-fat diet-fed rats were significantly increased when compared with the control group. Nevertheless, KD also markedly inhibited the expressions of serum cholesterol, triglyceride and LDL-C in high-fat diet-fed rats. Research has shown that HDL-C has the capacity to promote the elimination of cholesterol to prevent metabolic disorders. In our study, we observed that KD reversed the decrease in HDL-C expression in high-fat diet-fed rats. Consistent with the results of animal experiments, KD also prevented lipid accumulation and intercellular triglyceride and LDL-C levels in oleic acid-induced LO2 cells. Taken together, these results suggest that KD can prevent obesity and hyperlipidemia in high-fat diet-fed rats.

Current evidence suggests that insulin resistance plays a critical role in the pathogenesis of metabolic syndrome (Adnan et al., 2019). Therefore, enhancing insulin sensitivity is widely considered as an effective treatment strategy for metabolic syndrome. Impaired glucose tolerance is the most conspicuous hallmark of insulin resistance. Results of OGTT and ITT showed that a high-fat diet had successfully induced insulin resistance in rats. After 6 weeks of KD treatment, the glucose tolerance of high-fat diet-fed rats was considerably improved. Furthermore, we found KD could evidently inhibit HOMA-IR and increase insulin sensitivity in high-fat diet-fed rats.

The liver is the most essential organ regulating glycometabolism and lipid metabolism. Hepatic insulin resistance is a pivotal risk factor for the onset and progression of metabolic syndrome. Accordingly, human hepatic cell lines LO2 cells were used to explore the molecular mechanism of KD in the treatment of metabolic syndrome. In our study, we observed that KD distinctly enhanced glucose uptake and consumption in oleic acid-induced LO2 cells, suggesting an increase in insulin sensitivity. In conclusion, enhancing insulin sensitivity may be a critical mechanism for KD to prevent metabolic syndrome. According to the result of network pharmacology and experimental results *in vitro* and *in vivo*, we confirm the role of insulin resistance in the treatment of metabolic syndrome by KD. However, more work still needs to be done to verify the active ingredients and molecular mechanisms of KD in the treatment of metabolic syndrome revealed by network pharmacology (Li, 2021).

More and more evidence has confirmed the causality between autophagy deficiency and the pathogenesis of metabolic syndrome (Hyejin et al., 2018). Autophagy, a conservative catabolic process, can degrade excessive fatty acids and damaged organelles by lysosome to maintain cellular energy homeostasis. It is well documented that autophagy deficiency can induce metabolic syndrome by causing endoplasmic reticulum stress and mitochondrial dysfunction (Mizushima and Komatsu, 2011; Hyejin et al., 2018). Accordingly, plentiful autophagy activators have been employed to treat metabolic syndrome (Kim et al., 2014; Lim et al., 2014). The findings in our study are in line with previous reports that the enhancement of autophagic activity may be a novel therapeutic approach for metabolic syndrome. In our study, we found that KD could activate autophagic activity to improve the metabolic profile of oleic acid-induced LO2 cells and high-fat diet-fed rats. 3-MA, an autophagy inhibitor, could aggravate oleic acid-induced insulin resistance in LO2 cells by preventing KD-promoted autophagic activity. As we expect, RAP, an autophagy activator, could cooperate with KD to enhance autophagic activity to further improve oleic acid-induced insulin resistance in LO2 cells. These findings are confirmed by silencing Atg7 gene using small interfering RNA, which significantly reverses the therapeutic effect of KD on metabolic syndrome, which were manifested by aggravated insulin resistance and lipid accumulation in oleic acid-induced LO2 cells. Accordingly, it is concluded that the enhancement of autophagic activity may be the key molecular mechanism of KD in the treatment of metabolic syndrome.

## Conclusion

Our results demonstrate that KD improves metabolic syndrome via activating hepatic autophagy *in vivo* and *in vitro*. Our findings also suggest that KD can be considered as a complementary and alternative therapy for insulin resistance and metabolic syndrome.

## Data Availability

The raw data supporting the conclusions of this article will be made available by the authors, without undue reservation.
